# Epicardial adipose tissue thickness and NGAL levels in women with polycystic ovary syndrome

**DOI:** 10.1186/1757-2215-7-24

**Published:** 2014-02-16

**Authors:** Serap Baydur Sahin, Medine Cumhur Cure, Yavuz Ugurlu, Elif Ergul, Emine Uslu Gur, Nese Alyildiz, Mehmet Bostan

**Affiliations:** 1 Department of Endocrinology and Metabolism Disease, Recep Tayyip Erdogan University Medical School, Rize, Turkey; 2 Department of Biochemistry, Recep Tayyip Erdogan University Medical School, Rize, Turkey; 3 Department of Cardiology, Recep Tayyip Erdogan University Medical School, Rize, Turkey; 4 Department of Internal Medicine, Recep Tayyip Erdogan University Medical School, Rize, Turkey; 5 Department of Endocrinology and Metabolism Disease, Recep Tayyip Erdogan University Training and Research Hospital, 53020 Rize, Turkey

**Keywords:** Polycystic ovary syndrome, Epicardial adipose tissue thickness, NGAL

## Abstract

**Background:**

Polycystic ovary syndrome (PCOS) is associated with an increased cardiovascular disease (CVD) risk and early atherosclerosis. Epicardial adipose tissue thickness (EATT) is clinically related to subclinical atherosclerosis. In the present study, considering the major role of neutrophil gelatinase-associated lipocalin (NGAL) which is an acute phase protein rapidly releasing upon inflammation and tissue injury, we aimed to evaluate NGAL levels and EATT in PCOS patients and assess their relationship with cardiometabolic factors.

**Methods:**

64 patients with PCOS and 50 age- and body mass index-matched healthy controls were included in the study. We evaluated anthropometric, hormonal and metabolic parameters. EATT was measured by echocardiography above the free wall of the right ventricle. Serum NGAL and high-sensitive C- reactive protein (hsCRP) levels were measured by ELISA.

**Results:**

Mean EATT was 0,38 +/-0,16 mm in the PCOS group and 0,34 +/-0,36 mm in the control group (p = 0,144). In the obese PCOS group (n = 44) EAT was thicker compared to the obese control group (n = 41) (p = 0.026). Mean NGAL levels of the patients with PCOS were 101,98 +/-21,53 pg/ml, while mean NGAL levels were 107,40 +/-26,44 pg/ml in the control group (p = 0,228). We found a significant positive correlation between EATT and age, BMI, waist circumference, fasting insulin, HOMA-IR, triglyceride and hsCRP levels in PCOS group.

**Conclusions:**

Thickness of the epicardial adipose tissue can be used to follow the risk of CVD development in obese PCOS cases. However serum NGAL levels do not differ in patients with PCOS and control group.

## Introduction

Polycystic ovary syndrome (PCOS) is a common endocrine disorder that affects about 5-10% of women of reproductive age and characterized by hyperandrogenism, menstrual disturbance, chronic anovulation and infertility [1]. PCOS is frequently associated with multiple risk factors for coronary heart disease (CHD), including insulin resistance (IR), dyslipidemia, visceral obesity and hypertension [2,3]. It is reported to be associated with elevation of various markers of endothelial inflammation and abnormal endothelial function which plays a critical role in cardiovascular disease and in particular in the development and progression of atherosclerosis [4].

Neutrophil gelatinase-associated lipocalin (NGAL), also known as lipocalin-2 is an acute phase protein, that is rapidly released from neutrophils upon inflammation and tissue injury [5]. Additionally, it has been found to be expressed in several types of cells, including kidney tubular cells, adipocytes, macrophages, brain endothelial cells and hepatocytes [6,7]. NGAL has been implicated in some pathophysiological processes, including apoptosis, iron transport, inflammation, cell survival, tumorigenesis, and atherosclerosis [6,7]. Evidence suggests that NGAL plays an important role in systemic insulin sensitivity and glucose homeostasis [8,9]. In a recent study, NGAL was determined to reflect the degree of inflammatory process in coronary artery disease [10].

Epicardial adipose tissue is true visceral fat deposited around the heart, between the myocardium and visceral epicard [11]. It mediates atherosclerosis via expression of several bioactive molecules [12] and has been reported to reflect the intraabdominal visceral fat [13]. Epicardial adipose tissue thickness (EATT) has been demonstrated to correlate with the severity of coronary artery disease (CAD) and the extent of coronary artery atherosclerosis [14-16].

In this study, our aim was to measure NGAL levels and EATT in women with PCOS and compare them with those of age and body mass index (BMI)-matched controls and to evaluate their relationship with cardiometabolic factors.

## Materials and methods

### Study population

This study included 66 patients with PCOS and age- body mass index (BMI) matched 50 healthy controls. Women were all of the same ethnicity. All the patients gave a written consent. This study was approved by the Institutional Ethical Committee of the institution in which this study was conducted. All the patients were nonsmokers.

The diagnosis of PCOS was made according to the Rotterdam European Society for Human reproduction and Embryology/American Society for Reproductive Medicine– sponsored PCOS Consensus Workshop Group [17]. The revised diagnostic criteria of PCOS is as follows, with at least two of the following being required: i) Oligo and/or anovulation that is menstrual disturbance, ii) Clinical and/or biochemical signs of hyperandrogenism, iii) Polycystic ovarian appearance on ultrasound examination. Clinical hirsutism was defined by a modified Ferriman-Gallwey score of more than eight.

Patients and healthy subjects who had smoking history, diabetes mellitus, thyroid disorders, hyperprolactinemia, non-classical congenital adrenal hyperplasia (NCAH), androgen-secreting tumours, Cushing syndrome (1 mg dexamethasone suppression test), infection diseases, hypertension, hepatic or renal dysfunction were excluded from the study. The other exclusion criteria were; drug use within 3 months such as oral contraceptive agents, antilipidemic and hypertensive drugs, and insulin-sensitizing drugs. For the exclusion of NCAH, we measured 17OH-progesterone levels in the all patients. The patients who had basal 17OH-progesterone levels >4 ng/ml were diagnosed as NCAH. We performed ACTH stimulation test in patients who had >2 ng/ml 17-OHP levels. The diagnosis of NCAH was considered in patients with the poststimulation 17-OHP level exceed 10 ng/ml. These patients were not included in the study. We selected healthy volunteers from the subjects working at our hospital and students in our medical school and nursing school. Control group (n = 50) consisted of healthy patients who had hirsutism score <8, regular menses, every 21–35 days and normal androgen levels None of the women in the control group had polycystic ovary in ultrasound.

### Clinical, biochemical and hormonal measurements

Weight, height, waist circumference and systolic and diastolic blood pressure (BP) were measured. The BMI was calculated by dividing the body weight in kilograms by the square of the height in meters (kg/m^2^). Waist circumference was measured at the narrowest level between the costal margin and iliac crest. The BMI, waist circumference and hirsutism scores were assessed by a single investigator for all of the subjects.

Venous blood samples were obtained from all subjects following an 8–12 h overnight fast in the follicular phase of a spontaneous or progesterone induced menstrual cycle. The levels of fasting glucose, insulin, total cholesterol, triglyceride (TG), high-density lipoprotein cholesterol (HDL-cholesterol), low-density lipoprotein cholesterol (LDL-cholesterol) and high-sensitive C-reactive protein (hs-CRP) were measured. All the patients underwent a 75 gr oral glucose tolerance test (OGTT). An insulin resistance score Homeostasis Model Assessment-Insulin resistance (HOMA-IR) was computed by the following formula [18]: HOMA-IR = fasting plasma glucose (mmol/L) × fasting serum insulin (mU/mL)/22.5. The cut off value was taken 2.7 for HOMA-IR [19].

The hexokinase method was used to measure glucose levels, and photometric method (Abbott Architect c16000 otoanalyzer) was used to measure the total cholesterol, triglyceride (TG), high-density lipoprotein cholesterol (HDL-cholesterol), and low-density lipoprotein cholesterol (LDL-cholesterol) levels. Insulin was measured using CMIA (Chemiluminescent microparticle immunoassay) (Abbott, Architect system, USA). The concentration of high-sensitive C- reactive protein (hsCRP) was measured using immunotubidimetric method with Abbott Architect C16000 otoanalyzer (Abbott Diagnostic, USA). The cut off for hsCRP was taken <0.5 mg/dl.

Serum levels of insulin, follicle-stimulating hormone (FSH), luteinizing hormone (LH), prolactin, dehydroepiandrosterone sulfate (DHEAS), total testosterone, insulin and thyroid stimulating hormone (TSH) were measured using chemiluminescent microparticle enzyme immunoassay (CMIA) method with Abbott Architect i2000 (Abbott Diagnostic, USA). Serum 17 hydroxyprogesterone and free testosterone were measured by radioimmunoassay.

### Measurement of NGAL

The serum levels of NGAL were measured using enzyme-linked immunosorbent assay (ELISA) method. We used commercially available human NGAL ELISA kit (MyBiosource, USA). The procedure for the ELISA method was according to the instructions provided by the manufacturer. Absorbance was measured at a wavelength of 450 ηm using ELISA reader. The levels of NGAL are presented as ng/ml. The intra-assay and inter-assay coefficient of variation were 4.1% and 3.9%, respectively. The limit of detection (LOD) for the NGAL assay was <10 pg/ml.

### Measurement of epicardial adipose tissue thickness

All patients underwent 2D Doppler echocardiography with a Philips IE-33 system and S5-1 transducer (1e5 mHz, Philips, Bothell, WA, USA). The epicardial fat thickness (EFT) was identified as the echo-free space between the outer wall of the myocardium and the visceral layer of the pericardium, and its thickness was measured perpendicularly on the free wall of the right ventricle at end-systole in three cardiac cycles. Parasternal long- and short-axis views were used. The average value of three cardiac cycles from each echocardiographic view was considered [20,21]. All the measurements were done by the same cardiologist.

### Statistical analysis

Data were analyzed using SPSS Software (Version 17, SPSS, Inc., Chicago, IL, USA). Results were expressed as mean ± standard deviation. The Mann–Whitney U test was used to compare the continuous variables and the Chi-square test was used to compare categorical variables. Spearman’s rank correlation test was used for calculation of associations between variables. A p value of less than 0.05 was considered to be statistically significant.

## Results

We enrolled 66 patients with PCOS (mean age; 23,29 ±5,05 years, BMI; 32,09 ±9,80 kg/m^2^) and 50 age and BMI matched healthy control group (mean age; 22,02 ±5,05 years, BMI; 29,80 ±5,5 kg/m^2^) in the current study. The clinical and biochemical results of the study population are shown in Table 1.

**Table 1:** The clinical, biochemical and hormonal results in women with polycystic ovary syndrome (PCOS) patients and healthy controls

**Parameter**	**PCOS group**	**Control group**	***p***
	**(n = 66)**	**(n = 50)**	
Age (years)	23.29 ±5.05	22.02 ±5.05	0.183
BMI (kg/m2)	32.09 ±9.80	29.80 ±5.5	0.148
Waist circumference (cm)	8.67 ±19.37	92.28 ±13.30	0.048
Systolic blood pressure (mmHg)	126.77 ±14.15	113.22 ±9.99	0.0001
Diastolic blood pressure (mmHg)	76.18 ±11.37	68.20 ±6.74	0.0001
Fasting insulin (μIU/ml)	11.16 ±6.58	9.47 ±6.06	0.160
Fasting glucose (mg/dl)	97.30 ±13.97	89.10 ±10.56	0.001
HOMA-IR	2.71 ±1.7	2.16 ± 1.5	0.072
Total cholesterol (mg/dl)	194.81 ±39.55	173.28 ±29.99	0,002
Triglyceride (mg/dl)	115.91 ±58.18	93.30 ±39.30	0.020
HDL-C (mg/dl)	49.61 ±11.09	47.08 ±10.30	0.213
LDL-C (mg/dl)	122.83 ±34.43	107.0 ±24.64	0.007
hsCRP (mg/dl)	0.54 ±0.95	0.19 ±0.31	0.015
TSH (μIU/ml)	2.11 ±1.47	2.58 ±3.24	0.295
Prolactin (ng/ml)	18.3 ± 15.4	17.4 ± 6.9	0.590
Total testosterone (ng/ml)	0.85 ± 0.3	0.56 ± 0.1	0.0001
FSH (mIU/ml)	4.5 ± 1.2	4.5 ± 0.7	0.950
LH (mIU/ml)	5.7 ± 2.7	3.5 ± 0.5	0.0001
Estradiol (pg/ml)	43.9 ± 33.3	41.02 ± 3.1	0.536
DHEAS (μg/dl)	266.3 ± 123	203.85 ± 40.3	0.0001
Free testosterone (pg/ml)	3,3 ± 2.3	2.3 ± 0.4	0.008
EATT (mm)	0.38 ±0.16	0.34 ±0.36	0.144
NGAL (pg/ml)	101.98 ±21.53	107.40 ±26.44	0.228

Values are expressed as means ± SD. BMI body mass index, HOMA-IR homeostasis model assessment insulin resistance index, HDL-C high density lipoprotein cholesterol, LDL-C low density lipoprotein cholesterol, hs-CRP high-sensitive C- reactive protein, TSH thyroid stimulating hormone, FSH follicle-stimulating hormone, LH luteinizing hormone, DHEAS dehydroepiandrosterone sulfat, EATT epicardial adipose tissue thickness, NGAL neutrophil gelatinase-associated lipocalin.

Fasting plasma glucose, hsCRP, triglyceride, total and LDL cholesterol levels were significantly higher in women with PCOS (Table 1).

Mean EATT was 0,38 ±0,16 mm in the PCOS group and 0,34 ±0,36 mm in the control group (p = 0,144). Although EATT was higher in PCOS patients, the difference did not reach statistical significance. We found a significant correlation between EATT and age, BMI, waist circumference, fasting insulin, HOMA-IR, triglyceride and hsCRP in the patients with PCOS (Table 2). In the control group, EATT was also associated with.

**Table 2:** The correlation between epicardial adipose tissue thickness and metabolic parameters in patients with PCOS and control group

**Parameter**	**PCOS group (n = 66)**		**Control group (n = 50)**	
	**r**	**p value**	**r**	**p value**
Age	0,248	0,045^*^	0,231	0,106
BMI	0,641	0,0001^**^	0,314	0,027^*^
WC	0,563	0,0001^**^	0,258	0,070
FG score	0,061	0,626		
Fasting glucose	0,072	0,565	0,137	0,342
Fasting insulin	0,472	0,0001^**^	0,088	0,545
HOMA-IR	0,641	0,0001^**^	0,300	0,034^*^
Total cholesterol	0,216	0,081	0,299	0,035^*^
Triglyceride	0,363	0,003^**^	0,382	0,006^**^
LDL cholesterol	0,213	0,085	0,274	0,054
HDL cholesterol	-0,200	0,108	-0,243	0,093
hsCRP	0,324	0,008^**^	0,096	0,507
NGAL	0,086	0,494	-0,051	0,727
TSH	-0,110	0,377	0,010	0,946
Estradiol	0,026	0,834	0,029	0,846
LH/FSH	-0,367	0,068		
DHEAS	-0,133	0,286	-0,011	0,939
Total testosterone	0,185	0,137	0,078	0,596

r indicates Spearman’s rho correlation coefficient; *Correlation is significant at the 0.05 level. **Correlation is significant at the 0.01 level. a log transformed. BMI body mass index, HOMA-IR homeostasis model assessment insulin resistance index, HDL-C high density lipoprotein cholesterol, LDL-C low density lipoprotein cholesterol, hs-CRP high-sensitive C- reactive protein, NGAL neutrophil gelatinase-associated lipocalin, TSH thyroid stimulating hormone, FSH follicle-stimulating hormone, LH luteinizing hormone, DHEAS dehydroepiandrosterone sulfat.

Mean NGAL levels of the patients with PCOS were 101,98 ±21,53 pg/ml, while mean NGAL levels were 107,40 ±26,44 pg/ml in the control group (p = 0,228). Serum NGAL levels were not correlated with the metabolic parameters in both of the groups (Table 3).

**Table 3:** The correlation between NGAL levels and metabolic parameters in patients with PCOS

**Parameter**	**r**	**p value**
Age	0,042	0,738
BMI	0,149	0,235
WC	0,148	0,239
FG score	0,051	0,684
Fasting glucose	0,088	0,487
Fasting insulin	-0,109	0,880
HOMA-IR	0,031	0,808
Total cholesterol	0,013	0,917
Triglyceride	0,037	0,772
LDL cholesterol	0,019	0,882
HDL cholesterol	-0,081	0,523
hsCRP	0,064	0,612
EATT	0,086	0,494
TSH	0,176	0,162
Estradiol	-0,001	0,993
LH/FSH	-0,089	0,702
DHEAS	0,216	0,085
Total testosteron	-0,043	0,733

r indicates Spearman’s rho correlation coefficient. BMI body mass index, HOMA-IR homeostasis model assessment insulin resistance index, HDL-C high density lipoprotein cholesterol, LDL-C low density lipoprotein cholesterol, hs-CRP high-sensitive C- reactive protein, EATT epicardial adipose tissue thickness, TSH thyroid stimulating hormone, FSH follicle-stimulating hormone, LH luteinizing hormone, DHEAS dehydroepiandrosterone sulfat.

The patients with PCOS were divided into 2 groups according to the HOMA-IR levels. The cut-off point was 2.7 [22]. 28 PCOS patients had an HOMA-IR greater than 2.7, while 38 patients had an HOMA-IR lower than 2.7. EATT and hsCRP levels were higher in the insulin resistant group, however NGAL levels were similar in both of the groups (Table 4). The insulin resistant control group (n = 14) had also higher hsCRP levels, but EATT was similar.

**Table 4:** The comparison of PCOS and control group according to HOMA-IR levels

**Variable**	**HOMA-IR ≥ 2.7**	**HOMA-IR < 2.7**	***p***	**HOMA-IR ≥ 2.7**	**HOMA-IR < 2.7**	***p***
	**PCOS group**	**PCOS group**		**Control group**	**Control group**	
	**(n = 28)**	**(n = 38)**		**(n = 14)**	**(n = 36)**	
EATT (mm)	0.44 ± 1.1	0.34 ± 1.4	0.019	0.35 ± 0.16	0.33 ± 0.12	0.592
NGAL (pg/ml)	100.4 ± 23.2	103.1 ± 20.3	0.610	113.5 ± 21.4	105 ± 28.1	0.310
hsCRP (mg/dl)	0.89 ± 1.1	0.27 ± 0.6	0.009	0.38 ± 0.5	0.11 ± 0.14	0.004
BMI (kg/m2)	37.5 ± 10.3	28.1 ± 7.3	0.0001	34.06 ± 5.5	28.1 ± 4.6	0.0001

Values are expressed as means ± SD.

When we evaluate the PCOS patients according to the hsCRP values, 16 patients had hsCRP values higher than 0.5 mg/L and EATT was found to be higher in this subgroup (p = 0.007). In the control group, 4 patients had hsCRP levels ≥ 0.5 mg/L and EATT was similar in the PCOS and control group. NGAL levels did not differ in both of the groups according to hsCRP levels.

When we divide the PCOS patients according to BMI, 21 patients had BMI < 25 kg/m^2^. We compared the lean and obese patients with PCOS and found that NGAL and hsCRP levels were similar in both of the groups. However HOMA-IR and EATT was higher in the obese patients (p = 0.001, p = 0.007). In the control group, 41 patients had BMI > 25 kg/m^2^ and EATT, serum NGAL and hsCRP levels did not differ in the obese and healthy subjects (p = 0.902, p = 0.057, p = 0.342). When we compared the obese PCOS (n = 44) and obese control group (n = 41), EAT was thicker in the PCOS group (p = 0.026), however NGAL levels did not differ (Table 5).

**Table 5:** The comparison of obese PCOS and control subjects

	**Obese PCOS group**	**Obese control group**	***p***
	**(n = 44)**	**(n = 41)**	
EATT (mm)	0.42 ± 0.1	0.34 ± 0.1	0.026
NGAL (pg/ml)	104.2 ± 22.9	110.7 ± 27.1	0.235
HOMA-IR	3.1 ± 1.7	2.4 ± 1.5	0.045
hsCRP (mg/dl)	0.62 ± 0.9	0.21 ± 0.33	0.012

Values are expressed as means ± SD.

## Discussion

Our study shows that epicardial adipose tissue thickness is higher in obese PCOS patients compared with obese control subjects, however NGAL levels are similar in both of the groups. We demonstrated that EATT was positively correlated with age, BMI, waist circumference, fasting insulin, HOMA-IR, triglyceride and hsCRP levels. Therefore, EATT can be used to follow the risk of CVD development in obese PCOS cases.

Women with PCOS have multiple risk factors for cardiovascular disease (CVD) and is considered as a major long-term health risk. The risk factors for premature atherosclerosis and CVD, such as increased central adiposity, elevated blood pressure, higher total and LDL cholesterol levels, higher triglyceride levels, and higher plasma glucose levels were more prevalent in our PCOS population, compared with matched-for-age and BMI control women, as have been shown in previous studies [23,24]. These metabolic risk factors may predict the development of atherosclerosis in women with PCOS.

Although an increase in mortality due to coronary artery disease in patients with PCOS was not shown in the long term follow up [2], there is strong evidence that women with PCOS are at increased risk of metabolic cardiovascular syndrome [25]. In a study, coronary artery calcification was evaluated in younger women with PCOS 30–45 years as a measure of subclinical atherosclerotic disease burden, and it was found to be more prevalent in PCOS women [3]. In the other study, postmenopausal women noted 2.5 higher odds of obstructive coronary artery disease for those with clinical features of PCOS, even when controlling for metabolic and common cardiac risk factors [26].

Highly sensitive C-reactive protein (hsCRP) levels are good predictors of subclinical atherosclerosis and vascular events [22]. Many studies have also shown high hsCRP levels in women with PCOS [22,27] in contrast to others that say it is related only to obesity [28]. In our study, serum hsCRP levels were significantly higher in women with PCOS than the controls and it was correlated with BMI, EATT and decreased insulin sensitivity. Kim et al. have reported higher hsCRP levels even in lean PCOS women [22]. In our study, hsCRP levels did not differ in lean PCOS and lean control subjects.

NGAL is highly expressed in adipocytes, that its expression is regulated by obesity, and that it induces insulin resistance [8]. Studies investigating the relationship between NGAL levels and PCOS found confusing results. Cakal E et al. demonstrated that NGAL levels were higher in PCOS patients and was related with insulin resistance [29]. However, in the other study, NGAL levels were found to be lower in PCOS patients [30]. In our study, NGAL levels were similar in PCOS patients and healthy subjects. NGAL measurements also did not differ in groups based on the HOMA-IR and hsCRP levels and BMI.

In the recent studies it was found that serum levels of NGAL were significantly elevated in patients with angiographically confirmed CAD compared to those with normal arteries or controls [10,31,32]. The authors explain that these diffrences can result because of the expression of NGAL from vascular cells during atherogenesis. In our study, NGAL levels did not correlate with the cardiometabolic risk factors.

Epicardial adipose tissue thickness is clinically related to abdominal visceral adiposity which has significant impact on cardiovascular diasease risk [13]. In recent studies, EATT was found to be associated with coronary heart diseases [14-16].

There are only few studies evaluating the EATT in PCOS patients and in both of the studies EAT was thicker in the PCOS patients [33,34]. In our study, EAT was thicker in the PCOS group, but the difference was not significant. EATT was associated with the cardiometabolic risk factors, such as HOMA-IR and triglyceride in PCOS and control group. EATT was also associated with hsCRP in the PCOS patients, while it was not in the control group. When we compared the PCOS and control group according to HOMA-IR, hsCRP levels and BMI, we showed that EAT was thicker in the PCOS group who were obese and had higher HOMA-IR and hsCRP levels. However we did not demonstrate such a relationship in the control group. Additionally, in obese PCOS patients EATT was higher compared to obese healthy subjects. All obese subjects, whether PCOS or controls had similar EATT compared to the lean subjects. Therefore, we suggest that obesity does not solely influence EATT.

In conclusion, measurement of epicardial adipose tissue thickness is a non-invasive tool and easy to perform and appears to be a good method to follow the risk of cardiovascular disease development in obese and insulin resistant PCOS patients.

## Competing interests

The authors declare that they have no competing interests.

## Authors’ contributions

SBS, MCC and MB conceived the study and participated in its design. YU and EE performed the ultrasound examinations. SBS, EUG, NA and MB participated in the analysis and interpretation of data. SBS, NA and EUG collected the data. SBS, MCC, YU, EE, EUG, NA and MB wrote the paper. All authors read and approved the final manuscript.
